# Dental problems delaying the initiation of interferon therapy for HCV-infected patients

**DOI:** 10.1186/1743-422X-7-192

**Published:** 2010-08-17

**Authors:** Yumiko Nagao, Michio Sata

**Affiliations:** 1 Department of Digestive Disease Information & Research, Kurume University School of Medicine, Kurume, Fukuoka, 830-0011, Japan; 2 Division of Gastroenterology, Department of Medicine, Kurume University School of Medicine, Kurume, Fukuoka, 830-0011, Japan

## Abstract

**Background:**

There has been little discussion about the importance of oral management and interferon (IFN) therapy, although management of the side effects of therapy for chronic hepatitis C has been documented. This study determined whether dental problems delayed the initiation of IFN therapy for hepatitis C virus (HCV)-infected patients.

**Results:**

We analyzed 570 HCV-infected patients who were admitted to our hospital from December 2003 to June 2010 for treatment consisting of pegylated IFN (Peg-IFN) monotherapy or Peg-IFN/ribavirin combination therapy. The group comprised 274 men and 296 women with a mean age 57.2 years. Of the 570 patients, six could not commence Peg-IFN therapy, despite their admission, because of dental problems such as periodontitis, pupitis, and pericoronitis. The ages of six whose dental problems delayed the initiation of Peg-IFN ranged from 25 to 67 years, with a mean age of 47.3 ± 15.2 years. IFN therapy was deferred for 61.3 ± 47.7 days. Among the six subjects for whom IFN treatment was delayed, only one had a salivary flow that was lower than the normal value.

**Conclusions:**

Treatment of dental infections is required before IFN therapy for HCV infection can be started. To increase the depth of understanding of oral health care, it is hoped that dentists and medical specialists in all areas will hold discussions to generate cooperation.

## Background

In Japan, hepatocellular carcinoma (HCC) is the fourth leading cause of death in males and the sixth in females according to a recent survey. The incidence of HCC has increased in Japan throughout the past several decades [1]. Hepatitis C virus (HCV) is the major cause of HCC in Japan, with 70% of cases being HCV-related. It is assumed that between one and two million Japanese people are chronically infected with HCV [1].

Interferon (IFN) therapy for chronic hepatitis C is the only treatment for completely eliminating the virus. Combination therapy with pegylated IFN (Peg-IFN) and ribavirin has been recommended widely as the first choice for chronic hepatitis C patients with high viral loads. The sustained virological response (SVR) rate after 48 weeks of treatment at a standard dose is approximately 40 to 50% [2-5]. It has been shown that IFN therapy decreases the rate of development of HCC and improves the long-term prognosis [6-9].

Although IFN therapy has therapeutic benefits, the treatment produces a number of well-described side effects that are dominated by fatigue, influenza-like syndrome and neuropsychiatric symptoms [2-5,10-12] and management of such side effects is required during therapy. Among the side effects in a Japanese Phase III trial of Peg-IFN alfa-2a/alfa-2b and ribavirin, dental problems have been documented in patients with chronic hepatitis C. Meanwhile, it has been reported that hepatitis C infected patients have significant oral health needs [13-16] and that experience of dental caries is significantly worse for HCV-infected patients than patients in general [13].

Therefore, in the present study, we determined whether dental problems delayed the initiation of IFN therapy for HCV-infected patients.

## Methods

### Patients

A total of 570 HCV-infected patients who admitted to the Kurume University Hospital from December 2003 to June 2010 for treatment with Peg-IFN monotherapy or Peg-IFN/ribavirin combination therapy were studied (Table 1). The 570 patients were 274 men and 296 women with a mean age of 57.2 ± 11.6 years. They were consulted by one oral surgeon for each patient about presence of oral infection before commencing IFN treatment. All HCV-infected patients treated with IFN therapy at our hospital were required to undergo hospitalization for two weeks for therapeutic management and education about liver diseases.

**Table 1:** Characteristics of 570 patients

Men/Women		274/296	
Age (mean ± SD) years		57.2 ± 11.6	

Liver disease	AH-C	1	(0.2%)
	
	CH-C	471	(82.6%)
	
	CH-(B+C)	3	(0.5%)
	
	CH-C and post HCC treatment	20	(3.5%)
	
	LC-C	45	(7.9%)
	
	LC-C and post HCC treatment	30	(5.3%)

Peg-IFN therapy	Peg-IFN alfa-2a monotherapy	104	(18.2%)
	
	Peg-IFN alfa-2a monotherapy and trial	1	(0.2%)
	
	Peg-IFN alfa-2a/RBA	14	(2.5%)
	
	Peg-IFN alfa-2a/RBA and trial	5	(0.9%)
	
	Peg-IFN alfa-2b/RBA	438	(76.8%)
	
	Peg-IFN alfa-2b/RBA→Peg-IFN alfa-2a monotherapy	4	(0.7%)
	
	Peg-IFN alfa-2b/RBA→Peg-IFN alfa-2a monotherapy→Peg-IFN alfa-2a/RBA	1	(0.2%)
	
	Peg-IFN alfa-2b/RBA→Peg-IFN alfa-2a monotherapy→Peg-IFN alfa-2b/RBA	1	(0.2%)
	
	Peg-IFN alfa-2b/RBA→Peg-IFN alfa-2a/RBA	2	(0.4%)

HCV genotype	1a	2	(0.4%)
	
	1a or 1b	1	(0.2%)
	
	1b	401	(70.4%)
	
	2a	121	(21.2%)
	
	2b	24	(4.2%)
	
	3a	1	(0.2%)
	
	combination (1a and 1b)	1	(0.2%)
	
	combination (1b and 2b)	1	(0.2%)
	
	combination (1b and 3a)	1	(0.2%)
	
	combination (2a and 2b)	2	(0.4%)
	
	indeterminable	3	(0.5%)
	
	untested	12	(2.1%)

CH-C: chronic hepatitis C, CH-(B+C): chronic hepatitis B and C, LC-C: liver cirrhosis, HCC: hepatocelular carcinoma, Peg-IFN: pegylated interferon, RBV: ribavirin

We determined whether dental problems delayed the initiation of IFN therapy for these patients. Patients who underwent Peg-IFN therapy during dental treatment were excluded. Informed consent was obtained from all patients after the purpose and methods of the study were explained.

### Salivary flow

We used a simple and low-cost test for xerostomia detection, which requires chewing on a piece of gauze for 2 min. The results from 531 of 570 patients were quantified using the Saxon test. A salivary flow rate ≤ 2 g/2 min was judged as decreased salivary secretion.

### Serological assays

Serum samples were examined for the presence or absence of markers of HCV and HBV infection. The HCV RNA level before IFN therapy was analyzed by quantitative PCR assay (COBAS AMPLICOR HCV MONITOR v 2.0 Test, COBAS AmpliPrep/COBAS TaqMan HCV Test, Roche Molecular Systems, New Jersey, US) [17,18]. HCV genotype was determined by polymerase chain reaction assay, using a mixture of primers for the subtype, as reported previously [19].

Therapeutic response was judged after IFN therapy as: SVR - normalization of alanine aminotranferase (ALT) levels and HCV RNA negative for six months or more after treatment; transient response (TR) - normalization of ALT levels and undetectable HCV RNA during IFN treatment but HCV RNA-positive after IFN treatment; non-responder (NR) - neither normal nor negative results for six months or more.

As shown in Table 1, chronic hepatitis C with HCV genotype 1b was the most common. Patients with genotypes 2a/2b underwent Peg-IFN monotherapy and those with genotypes 1a/1b, a combination of Peg-IFN and ribavirn.

## Results

### Dental problems delayed the initiation of IFN therapy

Of 570 patients with HCV-related liver diseases, we documented six whose dental problems delayed the initiation of Peg-IFN therapy. Their ages ranged from 25 to 67 years, with a mean age of 47.3 ± 15.2 years. There were two men and four women (Table 2). These six patients could not commence IFN therapy, despite their admission for this treatment, and their therapy was deferred for 61.3 ± 47.7 days. Patient no. 1 had an acute odontogenic periostitis, resulting from periapical inflammation of endodontic origin. This was treated successfully by nonsurgical endodontics and administration of antibiotics. Patient no. 2 had an acute alveolar abscess, resulting from periodontal disease. His four molars were extracted after local anti-inflammation treatment. Patient no. 3 had a periapical periodontitis of the right mandibular second molar. The molar was extracted. Patient no. 4 had multiple dental problems with pain. After extirpation of dental pulps and extraction of teeth, she received IFN treatment. Patient no. 5 had apical periodontitis with gingival abscess, consequently her teeth were endodontically treated. Patient no. 6 had trismus and painful swallowing caused by pericoronitis of her wisdom tooth and she had a high white blood cell count of 10,200/mm3 on the day of admission. All six patients received IFN treatment after their dental treatment was completed. Nobody suffered from diabetes mellitus. The outcome of the patients was classified into three groups: SVR (n = 4), TR (n = 1), and NR (n = 1).

**Table 2:** Characteristics of six patients whose dental problems delayed the initiation of IFN therapy

**No**.	Age	Sex	Liver Disease	HCV RNA	HCV genotype	Dental problems that delayed the initiation of Peg-IFN therapy	Period to onset of IFN treatment after dental therapy (days)	Underlying disease	IFN therapy	Effect of IFN treatment
1	50	F	CH-C	980 kIU/ml	1b	#1. Acute periostitis of the right maxilla, #2. Periapical periodontitis of the right maxillary first molar	49	Gallbladder polyp	Peg-IFN alfa-2b/RBA	TR

2	67	M	CH-C	3,940 kIU/ml	1b	#1. Acute alveolar abscess of bilateral mandibular molars, #2. Periodontal diseases of the right mandibular first and second molars, the left mandibular first molar, and the left maxilla first and second molars	105	Gastric ulcer	Peg-IFN alfa-2b/RBA	NR

3	36	M	CH-C	over 500 kIU/ml	1b	Periapical periodontitis of the right mandibular second molar	4	None	Peg-IFN alfa-2b/RBA	SVR

4	47	F	CH-C	43 kIU/ml	2a	#1. Pulpitis of the right maxillary first premolar, the left maxillary second premolar, and the right mandibular second premolar, #2. Tooth stumps of the left maxillary canine and second premolar, and the right mandibular first premolar, #3. Dental caries of the right maxillary lateral incisor	97	Hypertension, Adjustment disorder, Gallstone	Peg-IFN alfa-2a	SVR

5	59	F	LC-C	471 kIU/ml	2a	#1. Periapical periodontitis and gingival abscess of the right mandibular lateral incisor, #2. Dental caries of bilateral mandibular central incisors	105	Depression, Hypertension, Osteoarthritis of the spine, Esophageal varices	Peg-IFN alfa-2b/RBA	SVR

6	25	F	CH-C	6.2 logIU/mL	1b	#1. Pericoronitis of the right mandibular wisdom tooth, #2. Horizontal impacted wisdom teeth of bilateral mandibles	8	None	Peg-IFN alfa-2b/RBA	SVR

CH-C: chronic hepatitis C, LC-C: liver cirrhosis, Peg-IFN: pegylated interferon, RBV: ribavirin,

TR: transient biochemical responders, NR: nonresponder, SVR: sustained virological response

### Salivary flow

The level of total saliva production, measured using the Saxon test, was 4.26 ± 1.91 g/2 min. The salivary flow rate was below the normal value in 54 patients (10.2%). Among the six subjects for whom IFN treatment was delayed, only one had a salivary flow that was lower than the normal value.

## Discussion

The results indicate that oral health care may be required before HCV-infected patients undergo IFN therapy. In our study, dental problems delayed the initiation of IFN therapy for a maximum of 105 days. HCV-infected patients treated with IFN therapy should be managed by intensive oral care because of lower resistance to infection during the therapy.

Poor of oral health has been reported for HCV-infected patients [13-16]. Coates et al. reported that the dental caries experience of HCV-infected subjects was significantly worse than that of patients in general, that the number of teeth missing from patients with hepatitis C infection also was significantly higher than for patients in general, and that periodontal health tended to be poor [13]. Griffin et al. found that patients with rheumatoid arthritis, diabetes or a liver condition were twice as likely to have an urgent need for dental treatment as patients who did not have these diseases and documented a high burden of unmet dental care needs among patients with chronic diseases [16]. The authors showed that HCV was the strongest predictor of patients reporting poor oral health.

Japanese HCV-infected patients tend to be older than those in other countries and their older age favors the onset of HCC, leading to an increased mortality rate [1]. Peg-IFN-ribavirin combination therapy is the standard treatment for chronic hepatitis C. Meanwhile, the frequency of adverse events in combination therapy is relatively high (20-64%) [2-5,10-12].

In a Japanese Phase III trial of Peg-IFN alfa-2a and ribavirin involving 199 patients with chronic hepatitis C, including 99 patients with IFN treatment-naive genotype 1 and 100 patients with patients whom had not had a SVR after IFN therapy, the oral side effects were: gingival bleeding and gingival swelling (6%), toothache (4.5%), gingivitis and periodontitis (3%), dental caries (1.5%), stomatitis and cheilitis (19.1%), disorder of taste (15.6%), dry mouth (6.5%), glossalgia and glossitis (4.5%), perioral paresthesia (2.5%), oral pain (0.5%), oral mucosal damage (0.5%), oral lichen planus (0.5%), oral hemorrhage (0.5%), dry lip (0.5%), and bulla of lip (0.5%). On the other hand, in a Japanese Phase III trial of Peg-IFN alfa-2b and ribavirin involving 332 chronic hepatitis C patients, including 269 patients for 48 weeks treatment duration with genotype 1b and high virus load, and 63 patients for 24 weeks treatment duration with others, oral side effect were: dental pulpitis, gingivitis, and periodontitis (8.9%), toothache (7.1%), dental abnormity (1.1%), stomatitis and cheilitis (26.8%), disorder of taste (26.8%), dry mouth (15.6%), glossitis (5.9%), oral discomfort feeling (2.6%), oral hemorrhage (0.4%), oral pain (0.4%), dry tongue (0.4%), decreased secretion of saliva (0.4%).

These findings indicate that dental management of HCV-infected patients is required before IFN therapy. However, in Japan the importance of oral health is often overlooked in HCV-infected patients and has not been discussed in detail up to now.

Several studies have shown an association between HCV and sicca symptoms [20,21]. Patients with chronic HCV infection also have been reported to be at a greater risk of developing insulin resistance [22,23]. Severe periodontal disease causes insulin resistance [24]. The reasons that HCV-infected individuals had problems such as dental caries and oral health care may include a decreased salivary flow rate, elicitation of periodontal disease by insulin resistance and difficulties for radical dental treatment of patients with liver disease who may have problems such as prolonged bleeding.

Henderson et al. reported HCV-infected cases and suggested the possibility of occasional discrimination by practitioners. They concluded that more effective oral health education is required for HCV-infected patients and dental practitioners [15]. We distributed a questionnaire to 209 patients who visited our hospital for liver disease treatment to determine whether patients with HCV or hepatitis B virus (HBV) disclosed their disease status to the personnel in dental clinics. We found that 59.8% always did so, 12.0% sometimes did so and 28.2% never did so. The main reason for nondisclosure was failure of dental healthcare workers to ask whether patients had systemic disease. Other reasons included fear of negative reactions from healthcare workers and not wanting dentists or staff to know their specific liver ailment [25]. To increase the depth of understanding of oral health care, it is hoped that dentists and medical specialists in all areas will hold discussions to create cooperation.

## Conclusions

In conclusion, the results of this study show that the treatment of dental infection is required before IFN therapy for HCV infection. On the basis of our results, we introduced systems in our hospital from November 2009 to ensure complete dental treatment before IFN therapy. We should enhance mutual understanding of various issues related to HCV-infected persons between the patient and the physician.

## Abbreviations

HCV: hepatitis C virus; HCC: hepatocellular carcinoma; IFN: interferon; Peg-IFN: pegylated IFN; SVR: sustained virological response; TR: transient response; NR: non-responder

## Competing interests

The authors declare that they have no competing interests.

## Authors' contributions

YN carried out most of the data collection and drafted the manuscript. MS contributed to data analysis. All authors read and approved the final manuscript.
